# New Appliances and Things Medical

**Published:** 1904-09-17

**Authors:** 


### NEW APPLIANCES AND THINGS MEDICAL.

### ALLENBURYS' THROAT PASTILLES.

(Allen and Hanburys, Ltd, London.)

We have received some samples of Messrs. Allen and Hanburys' well-known throat pastilles. These manufacturers make no less than sixty-two varieties of these pastilles, almost every possible astringent, sedative, and antiseptic being made up either singly or combined in this form. The pastilles, speaking generally, have a pleasant taste, and are sent out in flat tin boxes, adapting themselves very well to being carried in the pocket. Thirteen of these pastilles contain menthol, nine contain cocaine; the remainder are very varied in composition, containing heroin, morphine, eucaine, chlorate of potash, eucalyptus, borax, lettuce, etc. It need hardly be said that the pastilles are most useful and will continue to recommend themselves strongly to the medical practitioner.

